# First-Line LV5FU2 with or without Aflibercept in Patients with Non-Resectable Metastatic Colorectal Cancer: A Randomized Phase II Trial (PRODIGE 25-FFCD-FOLFA)

**DOI:** 10.3390/cancers16081515

**Published:** 2024-04-16

**Authors:** Jean-Louis Legoux, Roger Faroux, Nicolas Barrière, Karine Le Malicot, David Tougeron, Véronique Lorgis, Véronique Guerin-Meyer, Vincent Bourgeois, David Malka, Thomas Aparicio, Matthieu Baconnier, Valérie Lebrun-Ly, Joëlle Egreteau, Faïza Khemissa Akouz, Magali Terme, Côme Lepage, Valérie Boige

**Affiliations:** 1 Department of Hepato-Gastroenterology and Digestive Oncology, CHU d’Orléans, 14 avenue de l’Hôpital, CS 86709, 45067 Orleans CEDEX 2, France; 2 Department of Hepato-Gastroenterology and Digestive Oncology, Centre Hospitalier Les Oudairies, Boulevard Stéphane Moreau, 85925 La Roche sur Yon, France; roger.faroux@wanadoo.fr; 3 Department of Hepato-Gastroenterology and Digestive Oncology, Hôpital Européen, 6 Rue Désirée Clary, CS 70356, 13331 Marseille CEDEX 03, France; n.barriere@hopital-europeen.fr; 4 Fédération Francophone de Cancérologie Digestive (FFCD), EPICAD INSERM LNC-UMR 1231, Faculté de Médecine, University of Burgundy and Franche Comté, 7, Boulevard Jeanne d’Arc, 21079 Dijon, France; karine.le-malicot@u-bourgogne.fr; 5 Department of Hepato-Gastroenterology, CHU de Poitiers, 2 Rue de la Miletrie, BP 577, 86021 Poitiers, France; david.tougeron@chu-poitiers.fr; 6 Department of Medical Oncology, Institut de Cancérologie de Bourgogne, GRReCC, 18 Cours Général de Gaulle, 21000 Dijon, France; vlorgis@icb-cancer.fr; 7 Department of Medical Oncology, Institut de Cancérologie de l’Ouest, Boulevard Jacques Monod, 44805 Saint Herblain, France; veronique.guerin-meyer@ico.unicancer.fr; 8 Department of Hepato-Gastroenterology and Digestive Oncology, Centre Hospitalier Duchenne, Allée Jacques Monod-BP 609, 62321 Boulogne Sur Mer, France; v.bourgeois@ch-boulogne.fr; 9 Department of Cancer Medicine, Gustave Roussy, 114 rue Edouard Vaillant, 94805 Villejuif CEDEX, France; david.malka@imm.fr (D.M.); boige@igr.fr (V.B.); 10 Department of Gastroenterology, Saint Louis Hospital, APHP, Université Paris Cité, Paris, 1 Avenue Claude Vellefaux, 75475 Paris, France; thomas.aparicio@aphp.fr; 11 Department of Gastroenterology, Centre Hospitalier Annecy-Genevois, 1 Avenue de l’Hôpital, 74374 Pringy, France; mbaconnier@ch-annecygenevois.fr; 12 Department of Medical Oncology, CHU Dupuytren, 2 Avenue Martin Luther King, 87042 Limoges, France; valerie.ly@chu-limoges.fr; 13 Radiotherapy and Medical Oncology, Groupe Hospitalier Bretagne Sud, 5 Avenue de Choiseul, BP 12233, 56322 Lorient CEDEX, France; j.egreteau@ghbs.bzh; 14 Department of Hepato-Gastroenterology and Digestive Oncology, Saint Jean Hospital, 20 Avenue du Languedoc, BP 49954, 66046 Perpignan CEDEX 9, France; faiza.khemissa@ch-perpignan.fr; 15 INSERM U970—PARCC (Paris Cardiovascular Research Center), European Georges Pompidou Hospital, Université Paris Descartes, Sorbonne Paris Cité, 56 rue Leblanc, 75015 Paris, France; magali.terme@inserm.fr; 16 INSERM U866, Université de Bourgogne, 7 Boulevard Jeanne d’Arc, BP 27877, 21078 Dijon CEDEX, France; come.lepage@u-bourgogne.fr

**Keywords:** clinical trial, older patients, aflibercept, colorectal cancer, metastases

## Abstract

**Simple Summary:**

In this randomized phase II trial, which included 117 older patients with metastatic colorectal cancer receiving LV5FU2 regimen with or without aflibercept, the primary endpoint was 6-month progression-free survival (PFS). The clinical hypotheses expected a PFS rate at 6 months of over 40% (60% expected). It was 54.7% in both arms (90% CI 42.5–66.5 in both). Given the 6-month PFS, the study can be considered positive. However, the toxicity of aflibercept in this elderly population was high (grade ≥ 3 toxicity in 82% of patients versus 58.2% with LV5FU2 alone), and continuation of the trial into phase III is not envisaged.

**Abstract:**

Fluropyrimidine monotherapy is an option for some patients with inoperable metastatic colorectal cancer. Unlike bevacizumab, the addition of aflibercept, an antibody acting as an anti-angiogenic agent, has never been evaluated in this context. The aim of the study was to determine whether aflibercept could increase the efficacy of fluoropyrimidine monotherapy without increasing toxicity. This multicenter phase II non-comparative trial evaluated the addition of aflibercept to infusional 5-fluorouracil/folinic acid (LV5FU2 regimen) as first-line treatment in patients unfit to receive doublet cytotoxic chemotherapy. The primary endpoint was 6-month progression-free survival (PFS). The clinical hypotheses expected a PFS rate at 6 months of over 40% (60% expected). A total of 117 patients, with a median age of 81 years, were included: 59 in arm A (LV5FU2-aflibercept) and 58 in arm B (LV5FU2 alone). Six-month PFS was 54.7% in both arms (90% CI 42.5–66.5 in both). Median overall survival was 21.8 months (arm A) and 25.1 months (arm B). Overall toxicity was more common in arm A: grade ≥ 3 toxicity in 82% versus 58.2%. Given the 6-month PFS, the study can be considered positive. However, the toxicity of aflibercept in this population was high, and continuation of the trial into phase III is not envisaged.

## 1. Introduction

Colorectal cancers (CRCs) are among the leading cancers globally, being the third most frequent in men and the second in women worldwide [1], particularly in Western Europe (incidence of 41.2/100,000/year in men, 26.3 in women). In France, CRC is the most common cancer for both genders, with over 40,000 new cases annually [2]. The Francim network of French cancer registries reported that 33% of CRCs are metastatic or non-resectable at diagnosis [3]. Among the resected rectal and colon cancers, 30% and 22% will have a metastatic recurrence, respectively [4,5]. Therefore, it is estimated that about 50% of CCRs will develop metastases during their evolution. In France, these cancers mainly affect older individuals (median age 72 years in men, 75 years in women). Given the non-resectable and/or inoperable nature of these cancers (primary tumor and metastases), often due to patient age and co-morbidities, evaluating effective treatment strategies becomes crucial.

Two previous randomized studies demonstrated improved Progression-Free Survival (PFS) and Overall Survival (OS) in older metastatic colorectal (mCRC) patients by adding the anti-VEGF antibody bevacizumab to fluoropyrimidine (FP) monotherapy [6,7]. Two other randomized trials showed that adding bevacizumab to capecitabine [8], or miscellaneous chemotherapy in elderly patients in a phase II study (where PFS was primary objective) [9] did not improve OS; a second-line treatment was used in more than 50% of patients. A meta-analysis based on published data evaluated first-line monotherapy with FP alone versus combination treatment with oxaliplatin, irinotecan, or bevacizumab in elderly patients [10]. The addition of bevacizumab improved OS (HR = 0.78; CI = 0.63–0.96). It was dominated by the AVEX trial [6] (greater number of patients) and the Kabbinavar data [7]; however, in both series, the subsequent treatments were either not used or not specified. The treatment strategy was quite different from the current practice of sequential chemotherapy. Given the weakness of these results, it seemed justified to evaluate the benefit of adding another available anti-angiogenic agent such as aflibercept to 5FU monotherapy.

Aflibercept is a recombinant fusion protein, in which the human vascular endothelial growth factor (VEGF) extracellular receptor domain is fused with the Fc portion of human immunoglobulin G1 (IgG1). Aflibercept has already been used in combination with a simplified LV5FU2 regimen as part of the FOLFIRI regimen in the VELOUR trial [11], after a phase I trial recommended a dose of 4 mg/kg [12]. This trial demonstrated that the addition of aflibercept conferred a statistically significant survival benefit (PFS and OS) in patients with mCRC previously treated with oxaliplatin-based first-line chemotherapy, but failed to show a statistically significant increase in OS and PFS in patients over 65 years of age in an unplanned sub-group analysis [13]. In the AFFIRM study, a randomized phase II trial comparing fist-line mFOLFOX6 with or without aflibercept, median PFS was similar in both arms [14]. Median OS was 19 months in the aflibercept/mFOLFOX6 arm and 22 months in the mFOLFOX6 arm, showing no significant OS benefit in the aflibercept arm.

The aflibercept–LV5FU2 combination could be useful in mCRC patients with an indication for FP monotherapy, including patients with comorbidities and metastatic disease not amenable to curative treatment, as well as the frail and elderly [15,16]. The phase III study XELAVIRI (AIO KRK0110) recently reopened the question of first-line FP monotherapy strategy, with bevacizumab in both arms [17]. In the subgroup of patients older than 75 years [18], upfront doublet chemotherapy in combination with bevacizumab appeared to be deleterious. In this context, aflibercept in combination with 5FU, as compared with 5-FU alone, could possibly provide a survival benefit with little or no increase in adverse events.

Additionally, thymidylate synthase (TS) gene polymorphisms, influencing the clinical efficacy of 5-FU-based chemotherapy [19,20], were considered as a stratification factor in our randomization to ensure balanced arms.

The PRODIGE 25 trial was intended to study the safety and efficacy of the addition of aflibercept to 5FU monotherapy as the first-line treatment of mCRC in elderly patients with unresectable disease. A non-comparative randomized study was chosen to first evaluate the efficacy and toxicity of the combination in a small panel of patients before eventually conducting a larger phase III comparative study, in order to avoid exposing a large number of patients to a potentially toxic experimental treatment.

## 2. Materials and Methods

### 2.1. Study Design and Patients

This trial was a multicenter, randomized, non-comparative, open-label, phase II study. The main eligibility criteria were histologically proven non-resectable metastatic rectal or colon adenocarcinoma, not pre-treated for metastatic disease, patients’ age ≥ 65, performance status (PS) ≤ 2 according to World Health Organization (WHO) classification, and central determination of germline TS-5′UTR genotype on blood DNA for stratification. Inclusion and exclusion criteria can be found in Table S1.

### 2.2. Inclusion and Treatment

Standard investigations (biological, clinical, ECG) and a baseline tumor assessment (chest, abdomen, and pelvis CT-scan or chest CT-scan and hepatic or abdominal MRI) had to be performed within 21 days before randomization. Treatment was administered every 14 days as a simplified LV5FU2 regimen, preceded or not by an infusion of aflibercept of 4 mg/kg in one hour. Aflibercept (Zaltrap) is a product of SANOFI laboratory (82 Avenue Raspail, 94250 Gentilly, France), authorized in association with the FOLFIRI regimen after FOLFOX in patients with metastatic colorectal cancer. One hour after completion of the infusion, the simplified LV5FU2 regimen included folinic acid in IV (400 mg/m^2^ or 200 mg/m^2^ if L-folinic acid) in a 2 h infusion, with a 5 FU bolus in less than 10 min (400 mg/m^2^ in 100 cc glucose 50 mg/mL (5%)) and a continuous 5 FU infusion (2400 mg/m^2^ over 46 h).

The treatment was discontinued in the case of disease progression (radiological assessment every 8 weeks), death, withdrawal of consent, or unacceptable toxicity. Patients were randomized in a ratio of 1:1 using the minimization technique and considering the following stratification factors: center, age: ≤75 vs. >75, metastatic site (1 vs. >1), and TS-5′UTR polymorphism.

### 2.3. Study Objectives

The main objective of the trial was 6-month PFS (radiological progression according to RECIST 1.1 criteria or death) [21] after randomization, +/− 15 days according to the date of the nearest evaluation scanner and according to the investigator. Secondary endpoints were safety (toxicity according to NCI-CTC V4.0), overall survival (OS), the proportion of patients alive and without progression at 6 months (RECIST 1.1) according to central review, secondary curative resection rate at 1 year, and quality of life (using the EORTC QLQ-C30 evaluations [22]).

As a secondary objective, the comparison of PFS and OS according to TS polymorphism was planned on the entire patient population in the study, whether or not receiving aflibercept.

### 2.4. Statistical Analyses

The clinical hypotheses expected a PFS rate at 6 months of over 40% (60% expected). Using the Binomial Exact method and with a one-sided α risk of 5% and a power of 90%, 56 patients were needed per arm. Assuming that 5% of the patients could not be evaluated, 59 patients per arm were therefore to be randomized. The swoft program used for statistical results was SAS Software version 9.4 (SAS Institute, Cary, NC, USA). The main analyses were conducted on a modified intention-to-treat basis (mITT), meaning we included all randomized patients, whatever their eligibility criteria, who had received at least one dose of treatment. Baseline characteristics were described using descriptive statistics as percentages for categorical and ordinal variables, and means (with standard deviations) and medians (with inter-quartile and min–max intervals) for continuous variables. The results were presented by treatment group for the overall population and according to TS-5′UTR polymorphism. Per-protocol analyses were conducted in patients who received at least two courses of chemotherapy regardless of doses, i.e., two doses of aflibercept, and with a WHO status prior to treatment < 2; the per-protocol analysis was conducted according to the treatment actually received. Safety analyses were conducted on a safety population.

The proportion of patients alive and without progression at 6 months (PFS) was calculated according to the investigator’s evaluation (using RECIST v1.1) at 6 months (+/− 15 days). It was described using a percentage and a two-sided 90% confidence interval. PFS2 was the time between the date of the first second line (L2) round and the start of the third line (L3), the date of death, or of last news if alive. For survival analyses, censored data were estimated using the Kaplan–Meier method. The median times and percentages at different time points were reported with their 95% confidence intervals.

The number of treatment cycles, the dose received, and the percentages of dose received over the theoretical dose were described, as was the percentage of patients with at least one dose modification or at least one chemotherapy cycle delay.

PFS and OS were also analyzed according to TS-5′UTR polymorphism, as specified in the statistical analysis plan. Univariate and multivariate analyses were conducted on an exploratory basis for the main criterion and overall survival using, respectively, logistic regression and the Cox model.

Toxicity was described according to System Organ Class (SOC) and Preferred Term (PT) (NCI-CTC v4.0). Serious adverse events were analyzed by the Pharmacovigilance department. A statistical analysis plan was written and signed before the database was locked.

### 2.5. Ethics Approval

PRODIGE 25-FOLFA, sponsored by Fédération Francophone de Cancérologie Digestive (FFCD), was authorized in France by the Agence Nationale de Sécurité du Médicament et des Produits de la Santé (ANSM) on 5 September 2014 and by the Comité de Protection des Personnes (CPP) of Tours on 27 August 2014. The trial was registered on the clinical trials.gov website under number NCT02384759. The study complies with the Declaration of Helsinki and the principles of Good Clinical Practice guidelines. Informed consent was obtained prior to inclusion of each patient.

## 3. Results

**Figure 1:**
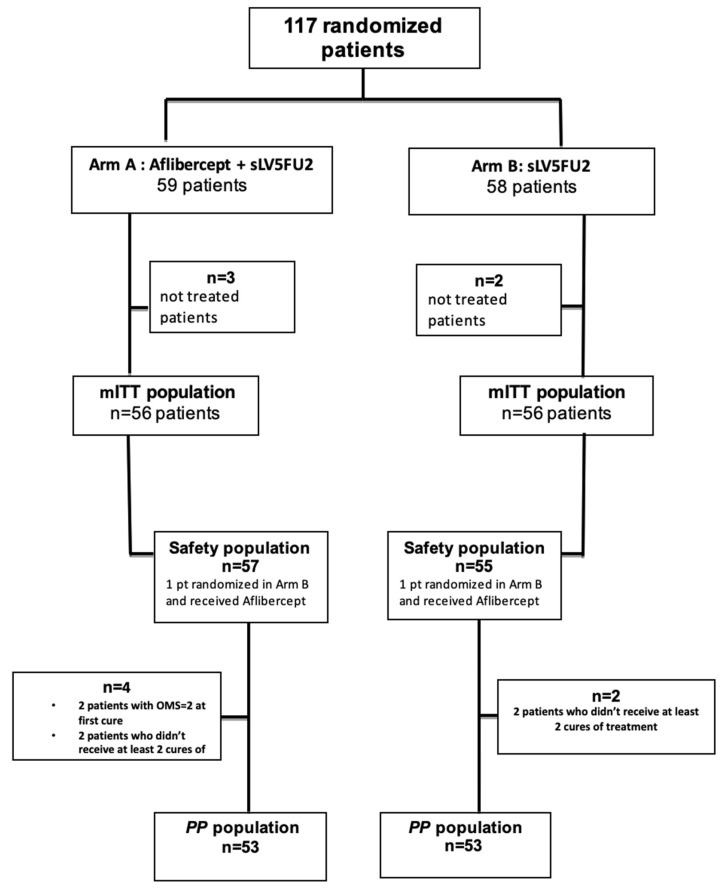
Study flow chart. mITT: modified intent-to-treat population. PP: per protocol population. sLV5FU2: simplified LV5FU2 regimen.

Between May 2015 and September 2020, 117/118 patients (pts) were included (Figure 1), with 59 in arm A (5FU-aflibercept) and 58 in arm B (5FU alone). The number of evaluable patients was reached given the length of follow-up, and inclusions were stopped in October 2020.

### 3.1. Population

Inclusion criteria were met in all pts, and a non-inclusion criterion was present in two pts in arm B (history of cancer). The distribution of pts according to the type of institution that managed them was similar in both arms: 34% of pts were being treated in a university hospital, 30% in a general hospital, 22% in a private clinic, and 15% in a cancer center.

The median age was 81 years (range 67–91), age was over 75 years in 81% of pts, and 61.5% were male. The main patient characteristics (Table 1) were well balanced between the two arms, except for a trend towards a higher WHO PS (*p* = 0.07) and Köhne score (*p* = 0.09) [23] as well as significantly higher alkaline phosphatase (ALP) (*p* < 0.02) and GGT (*p* < 0.04) levels in arm A. After primary tumor resection, 20% of patients received adjuvant chemotherapy. A trend towards a greater proportion of synchronous metastasis in arm A and peritoneal involvement in arm B was observed (Table 2).

### 3.2. Treatment Efficacy

**Figure 2:**
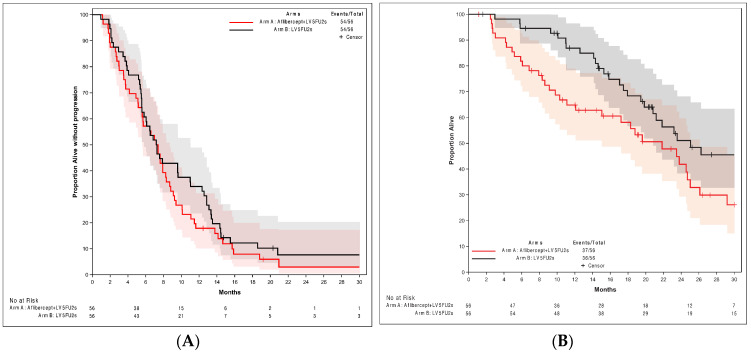
Survival curves for intent-to-treat (modified). (**A**) Progression-free survival (with 95% confidence interval). (**B**) Overall survival (with 95% confidence interval). LV5FU2s: simplified LV5FU2 regimen.

Six-month PFS was 54.7% in both arms (90% CI: 42.55; 66.47), exceeding the lower 90% CI limit of 40%. The primary endpoint was met. In the per-protocol population, PFS was similar in both arms: 7.4 months (90% CI: 5.59; 8.31) in arm A and 7.3 months in arm B (90% CI: 5.59; 11.01). No complete response was observed, but objective partial responses (as best response) were obtained in 28% (arm A) and 40% of pts (arm B). The disease control rate was 84% (arm A) and 89% (arm B). The PFS curves are shown in Figure 2. In the m-ITT analysis, OS rates were (arm A vs. B) 65% and 87% at 1 year and 42% and 51% at 2 years. Median OS was 21.8 months (CI 95%: 12.09; 25.03) and 25.1 months (CI 95%: 19.84; 31.93) in arms A and B, respectively. The OS curves are shown in Figure 2.

For exploratory purposes, univariate and multivariate analyses for PFS and OS were conducted, involving the following items: treatment arm, age (<75 vs. ≥75 years), number of metastatic sites (1 vs. >1), TS *5′UTR* polymorphism, Köhne score, WHO performance status, location of primary tumor (right colon/left colon/rectum), resection of primary tumor, adjuvant treatment, *RAS* and *BRAF* status (mutated, wild, not done). In the multivariate analyses, resection of the primary tumor was associated with better PFS at 6 months (OR = 0.60 [0.39; 0.92], *p* = 0.02) and a better OS (HR = 0.53 [0.30; 10.93], *p* = 0.027). The lower Khöne’s score was linked to a better OS (HR = 0.43 [0.19; 1], *p* = 0.050). The 2R2R TS-5′UTR polymorphism was associated with a better PFS at 6 months versus 3R/3R (OR = 0.26 [0.08; 0.82], *p* = 0.004).

### 3.3. Toxicity

As shown in Table 3 and Table 4, grade ≥ 3 toxicity was more frequent in arm A: 82% vs. 58% (*p* = 0.048) of patients. Grades 3–5 cardiovascular toxicity, including stroke, occurred in 58% of pts (arm A) vs. 29% (arm B). Aflibercept-induced toxicity included grade ≥ 3 hypertension (42% vs. 18% of pts), any grade proteinuria (51% vs. 11% pts), grade < 3 dysphonia (19% vs. 2%), and epistaxis (26% vs. 16%). One colon perforation occurred in arm A, and none in arm B. Hemorrhage seemed more frequent in arm A.

Aflibercept was stopped for various reasons, principally toxicity, in 36% of patients; LV5FU2 was stopped in nine patients (17%) vs. one (2%) in arms A and B, respectively. Aflibercept toxicity (in most cases, proteinuria, hypertension, or both) led to its temporary discontinuation in 13 pts (23%) and a dose decrease (of more than 25%) in 37% of patients. The mean dose-intensity was 73% (SD = 32.2). After permanent discontinuation of aflibercept, LV5FU2 was always continued. The dose-intensity of 5FU was similar in both arms: on average, 91% (SD = 12.1) in arm A and 94.8% (SD = 9.2) in arm B. A decrease > 25% of the 5FU bolus dose was decided in 25% (arm A) and 16% (arm B) of patients after a median of 29 days vs. 73 days following treatment initiation, but the dose intensity of the 5FU bolus was similar in the both arms: 78% (SD = 32) and 83% (SD = 31).

### 3.4. Follow-Up

Twenty-three patients in arm A (40%) vs. 39 (71%) in arm B (*p* = 0.001) received at least one subsequent line of treatment, 14% of pts vs. 29% who received two subsequent lines. Fourteen percent of patients in arm A vs. 29% in arm B received FOLFOX and FOLFIRI after the first line. The proportions of patients with subsequent FOLFOX or FOLFIRI and the administration of anti-angiogenics were similar in both arms. Seven percent of patients in arm A and 20% in arm B received anti-EGFr in further lines. Median PFS2 was 4.6 months (arm A) and 6.9 months (arm B). PFS2 at 12 months was, respectively, 8.7% (CI 1.5; 24.17) and 18.8% (CI 7.95; 33.16). Median survival in patients without L2 was 7 months in arm A and 20 months in arm B. Twelve-month survival without L2 was 43% (CI 24.24; 60.75) vs. 59% (CI 26.43; 80.84). Secondary resection (R0) was performed for the primary cancer in four pts (three in arm A), and for metastases in three patients (two in arm A). The only patient with both resections was in arm B.

## 4. Discussion

Our study showed that aflibercept combined with simplified LV5FU2 in elderly mCRC patients had an acceptable safety profile and met the primary endpoint in efficacy. However, these results are to be taken with caution, because results in LV5FU2 alone were similar.

### 4.1. Choice of the Trial Design

Aflibercept combined with fluoropyrimidine alone has not previously been evaluated. We chose the LV5FU2 regimen as the FP treatment, rather than capecitabine; the MRC FOCUS2 study [15], in elderly and frail pts, showed a higher risk of having any grade 3 or worse toxic effect with capecitabine than with fluorouracil (40% vs. 30%; *p* = 0.03). Infusion of 5FU, as in the LV5FU2 regimen, appeared less toxic than capecitabine in two meta-analyses [24,25], contrary to the results of the initial comparison with the Mayo Clinic bolus regimen [26,27]. In addition, many pts take concomitant medications, such as proton pump inhibitors, which interfere with capecitabine efficacy [28]. The simplified LV5FU2 regimen, despite an increased dose of 5FU but with less folinic acid (administered only on the first day), seems to be less effective (decrease of 4 months in OS) than conventional LV5FU2 [29], but it is currently more acceptable to physicians and patients, as the patient does not have to return to the hospital on the second day.

### 4.2. PFS

The results with bevacizumab combined with FP in AVEX [6], MAX [8], and XELAVIRI [17] studies were similar to our study results (Table 5) with regard to PFS. Nevertheless, PFS with capecitabine plus bevacizumab was better than that with capecitabine alone. This was not the case in our study. The trend towards differences between our two arms in some predictors of a poorer prognosis (higher WHO PS, Köhne score) as well as the higher ALP level in arm A do not seem sufficient to explain these results. In our population, results with LV5FU2 alone seemed better than those with capecitabine alone in the two studies above, better than LV5FU2 in the FFCD 2001-02 and the FOCUS trial [29,30]. All these studies began over 10 years ago, and it is possible that improved supportive care associated with chemotherapy could explain an improvement in survival.

### 4.3. OS

In the MAX study [8], the percentage of L2 was over 60% in both arms; the addition of bevacizumab in L1 did not significantly improve OS. In the AVEX [6] study, the level of doublet chemotherapy after progression was 8% only in the association arm and 4% in the monotherapy arm. This treatment strategy is therefore not the one that is currently implemented. In the FOLFA study, we observed a median OS consistent with the previous trials studying the bevacizumab–capecitabine combination (Table 5), but the LV5FU2 arm had particularly high OS. Could the greater efficiency of our “control” arm erase the difference with an arm penalized by its toxicity? The non-statistically significant trend towards higher PS status and Köhne score in arm A may suggest a discrete imbalance between the two arms and may have contributed to the difference in OS. OS was not statistically evaluated, as the trial was not designed to be comparative. The Köhne score was a prognostic criterion in multivariate analysis.

### 4.4. Toxicity

Toxicity was class-dependent, principally in high blood pressure and proteinuria, as in the VELOUR study, but lower than those in the AFFIRM [14] study with FOLFOX. In the VELOUR study, the treatment was discontinued after adverse events in 27% of patients vs. 12% (aflibercept arm vs. FOLFIRI alone arm). In the three studies, it was possible to stop aflibercept and continue the chemotherapy. In the VELOUR study, the chemotherapy dose was decreased more often in the aflibercept arm for irinotecan (37% vs. 23%) and for 5FU (39% vs. 22%). In the three studies, there was no plan to decrease the dose of aflibercept for toxicity. The dose-intensity of capecitabine in the AVEX and MAX studies was not reported. In our study, the dose-intensity of 5FU was similar in both arms, but the 5FU bolus was more often stopped, and stopped earlier, in the aflibercept arm. In addition, aflibercept was often discontinued or administered at a reduced dose because of toxicity. Its dose-intensity was 73.3%. It was not specified in the previous studies with FOLFIRI or FOLFOX. The fact that more patients in arm B of our study received subsequent treatment lines with FOLFOX and FOLFIRI and/or anti-EGFr antibodies cannot explain the trend towards improved OS in this arm; median survival for patients without L2 also seemed lower in the aflibercept arm.

### 4.5. Limits of the Study

The main limit of our study is the non-comparative design. This design was chosen to evaluate the efficacy of aflibercept with a reduced number of patients included and therefore less exposure to the potential toxicity of the drug. It did not allow us to use comparative statistical tests to evaluate the primary and secondary endpoints. The second limit of our study is the absence of data about the geriatric evaluations before inclusion. A geriatric assessment is recommended in France before any decision on chemotherapy for older patients [31]. Finally, there were missing data on BRAF mutations, which were not routinely tested in France at the start of the trial.

### 4.6. Place of Aflibercept in the Therapeutic Strategy

In the second-line setting (L2), the efficacy of the combination aflibercept–irinotecan-FP was demonstrated in the VELOUR trial, converging with a study with bevacizumab [32]. As a VEGF trap with a wider range of targets than bevacizumab, aflibercept may not be as effective as bevacizumab in L1; it could inhibit targets involved in bevacizumab resistance (VEGF-A and PIGF) and therefore be more effective in L2 [33]. This could explain the lower activity of aflibercept in L1, as in the AFFIRM study. However, the backbone chemotherapy in this study was FOLFOX, which could interfere with the results, as in studies with other anti-angiogenic agents [14]. There are no published results from randomized studies of aflibercept in combination with FOLFIRI in non-pre-treated patients. Furthermore, aflibercept may be less effective in older patients, as seen in our study and in the VELOUR trial. It would be interesting to consider a planned dose reduction of aflibercept after toxicity and in the elderly. This is not currently standardized. More generally, it would be interesting to gain a better understanding of the biomarkers of anti-angiogenic efficacy and toxicity and to better document biologically the specificities of elderly subjects.

## 5. Conclusions

Our study showed that aflibercept combined with LV5FU2 in elderly mCRC patients had an acceptable safety profile and met the primary endpoint in efficacy. However, caution is warranted, as the results in LV5FU2 alone were similar. The non-comparative design and potential confounding factors emphasize the need for careful interpretation, but the results of the FOLFA study do not support a randomized phase III trial evaluating first-line fluoropyrimidine with or without aflibercept in mCRC patients.

## Figures and Tables

**Table 1:** Patient characteristics.

		Arm A: Aflibercept + sLV5FU2 ^a^	Arm B: sLV5FU2 ^a^	Total
		(*n* = 56)	(*n* = 56)	(*n* = 112)
Age	*n*	59	58	117
	Moy. (SD)	80.27 (5.43)	80.23 (5.40)	80.25 (5.39)
	Médiane	80.99	80.68	80.87
	Q1; Q3	76.47; 83.68	78.07; 83.92	77.43; 83.76
	Min; Max	68.15; 90.49	67.05; 90.46	67.05; 90.49
	≤75 years	13 (22.0%)	9 (15.5%)	22 (18.8%)
	>75 years	46 (78.0%)	49 (84.5%)	95 (81.2%)
Number of metastatic sites	*n*	59	58	117
	1	23 (39.0%)	26 (44.8%)	49 (41.9%)
	>1	36 (61.0%)	32 (55.2%)	68 (58.1%)
TS-5UTR polymorphism	*n*	59	58	117
	2R2R genotype	17 (28.8%)	15 (25.9%)	32(27.4%)
	2R3R genotype	28 (47.5%)	29 (50.0%)	57 (48.7%)
	3R3R genotype	14 (23.7%)	14 (24.1%)	28 (23.9%)
BMI (Kg/m^2^)	*n*	56	56	112
	Median	24.84	26.12	25.56
	Q1; Q3	21.85; 28.15	23.50; 28.04	22.94; 28.04
	Min; Max	18.36; 36.51	17.36; 37.10	17.36; 37.10
WHO performance status	*n*	56	56	112
	0	14 (25.0%)	23 (41.1%)	37 (33.0%)
	1	42 (75.0%)	33 (58.9%)	75 (67.0%)
Systolic blood pressure (mmHg)	*n*	48	42	90
	Median	139.00	134.50	136.50
	Q1; Q3	124.00; 150.00	126.00; 145.00	125.00; 147.00
	Min; Max	103.00; 210.00	110.00; 188.00	103.00; 210.00
Diastolic blood pressure (mmHg)	*n*	47	42	89
	Median	70.00	74.50	73.00
	Q1; Q3	68.00; 80.00	70.00; 80.00	69.00; 80.00
	Min; Max	59.00; 110.00	60.00; 91.00	59.00; 110.00
Köhne score	*n*	56	56	112
	Low	22 (39.3%)	25 (44.6%)	47 (42.0%)
	Middle	27 (48.2%)	30 (53.6%)	57 (50.9%)
	High	7 (12.5%)	1 (1.8%)	8 (7.1%)
Creatinine clearance (mL/min)	*n*	56	56	112
	Median	65.0	67.5	66.5
	Q1; Q3	57.5; 81.0	56.0; 84.5	57.0; 82.5
	Min; Max	42.00; 130.00	42.00; 126.00	42.00; 130.00
Albumin (g/L)	*n*	53	53	106
	Median	38.00	40.00	39.00
	Q1; Q3	35.00; 41.00	38.00; 43.00	36.00; 41.00
	Min; Max	29.00; 45.00	22.00; 48.00	22.00; 48.00

^a^ sLV5FU2: simplified LV5FU2.

**Table 2:** Tumor characteristics.

		Arm A: Aflibercept + sLV5FU2 ^a^	Arm B: sLV5FU2 ^a^	Total	*p*-Value
		(*n* = 56)	(*n* = 56)	(*n* = 112)	
Time from diagnosis to randomization (months)	*n*	53	51	104	
	Median	1.94	1.81	1.89	
	Min; Max	0.62; 57.82	0.33; 38.28	0.33; 57.82	
Location of the primary tumor	*n*	55	55	110	
	Rectum	10 (18.2%)	13 (23.6%)	23 (20.9%)	
	Right colon	32 (58.2%)	31 (56.4%)	63 (57.3%)	
	Left colon	13 (23.6%)	11 (20.0%)	24 (21.8%)	
Resection of the primary tumor	*n*	56	56	112	
		31 (55.4%)	39 (69.6%)	70 (62.5%)	
Synchronous or metachronous metastases	*n*	50	51	101	
	Metachronous	15 (30.0%)	22 (43.1%)	37 (36.6%)	
	Synchronous	35 (70.0%)	29 (56.9%)	64 (63.4%)	
Hepatic metastases	*n*	56	56	112	
		43 (76.8%)	39 (69.6%)	82 (73.2%)	
Pulmonary metastases	*n*	56	56	112	
		30 (53.6%)	33 (58.9%)	63 (56.3%)	
Peritoneal metastases	*n*	56	56	112	
		8 (14.3%)	16 (28.6%)	24 (21.4%)	
Alkaline phosphatases (UI/L)	*n*	56	55	111	*W*: 0.02
	Median	126.00	96.00	106.00	
	Min; Max	54.00; 978.00	31.00; 442.00	31.00; 978.00	
GGT (UI/L)	*n*	56	54	110	*W*: 0.04
	Median	93.50	51.00	76.50	
	Min; Max	12.00; 1957.00	12.00; 868.00	12.00; 1957.00	
Lactate Dehydrogenase (UI/L)	*n*	48	52	100	
	Median	250.50	301.50	283.00	
	Min; Max	115.00; 2083.00	133.00; 1129.00	115.00; 2083.00	
*RAS* status	*n*	56	56	112	
	Wild	24 (42.9%)	17 (30.4%)	41 (36.6%)	
	Mutated	24 (42.9%)	34 (60.7%)	58 (51.8%)	
	Not done	8 (14.3%)	5 (8.9%)	13 (11.6%)	
*BRAF* status	*n*	56	56	112	
	Wild	38 (67.9%)	36 (64.3%)	74 (66.1%)	
	Mutated	6 (10.7%)	3 (5.4%)	9 (8.0%)	
	Not done	12 (21.4%)	17 (30.4%)	29 (25.9%)	

^a^ sLV5FU2: simplified LV5FU2.

**Table 3:** Toxicities: maximal grade per patient; number (percentage).

	Arm A: Aflibercept + sLV5FU2 ^a^		Arm B: sLV5FU2 ^a^	
	**Grade 1/2**	**Grade 3/4/5**	**Grade 1/2**	**Grade 3/4/5**
	(*n* = 57)	(*n* = 57)	(*n* = 55)	(*n* = 55)
At least one toxicity	57 (100.0)	47 (82.5)	55 (100.0)	32 (58.2)
Obstruction or sub-obstruction	1	3		3
Digestive perforation		1 (1.8)		
Hemorrhage	3 (5.3)	2 (3.5)	1 (1.8)	
**Hematological toxicity**				
Anemia	35 (61.4)	2 (3.5)	41 (74.5)	1 (1.8)
Neutropenia	7 (12.3)	2 (3.5)	13 (23.6)	3 (5.5)
Thrombopenia	16 (28.1)		15 (27.3)	
**Cardiovascular toxicity (all)**	**22 (38.6)**	**33 (57.8)**	**20 (36.4)**	**16 (29.1)**
Rhythm disorders	2 (3.6)		2 (3.6)	
Thoracic pain			1 (1.8)	1 (1.8)
Cardiac insufficiency				1 (1.8)
Unspecified tromboembolic event	1 (1.8)	1 (1.8)		
Arterial tromboembolic event	2 (3.5)		1 (1.8)	
Venous tromboembolic event	1 (1.8)	4 (7.0)	4 (7.3)	2 (3.6)
Peripheral ischemia			1 (1.8)	
Stroke		3	1	1
Hematoma				1 (1.8)
Hypertension	13 (22.8)	24 (42.1)	10 (18.2)	10 (18.2)
Hypotension	3 (5.3)	1 (1.8)		
**Renal toxicity**				
Proteinuria	22 (38.6)	7 (12.3)	6 (10.9)	
Increased creatinine	22 (38.6)	1 (1.8)	21 (38.2)	
**Other toxicities**				
Palmar-plantar erythrodysthesia syndrome	15 (26.3)	3 (5.3)	12 (21.8)	1 (1.8)
Oral mucositis	24 (42.1)	1 (1.8)	23 (41.8)	1 (1.8)
Nausea	19 (33.3)		22 (40.0)	
Headache	7 (12.3)	1 (1.8)	1 (1.8)	
Peripheral sensory neuropathy	5 (8.8)		1 (1.8)	
Alteration of voice	11 (19.3)		1 (1.8)	
Epistaxis	14 (24.6)	1 (1.8)	9 (16.4)	
Cough	8 (14.1)		3 (5.5)	
Weight loss	9 (15.8)	1 (1.8)	4 (7.3)	1 (1.8)
Anorexia	29 (50.9)	3 (5.3)	15 (27.3)	2 (3.6)
Fatigue	41 (71.9)	9 (15.8)	37 (67.3)	6 (10.9)
Hyperkalemia	18 (31.6)		5 (9.1)	

^a^ sLV5FU2: simplified LV5FU2.

**Table 4:** Overall toxicity. A: maximal grade per patient in the overall population; B: maximal grade per patient for men; C: maximal grade per patient for women.

A				
		Arm A: Aflibercept + sLV5FU2 ^a^	Arm B: sLV5FU2 ^a^	*p*-Value
		(*n* = 57)	(*n* = 55)	
NCI-CTC classification: maximal grade per patient	*n*	57	55	*p* = 0.0356
	1	2 (3.5%)	2 (3.6%)	
	2	8 (14.0%)	21 (38.2%)	
	3	39 (68.4%)	26 (47.3%)	
	4	4 (7.0%)	5 (9.1%)	
	5	4 (7.0%)	1 (1.8%)	
**B**				
		**Bras A: Aflibercept + sLV5FU2 ^a^**	**Bras B: sLV5FU2 ^a^**	***p*-value**
		(*n* = 36)	(*n* = 32)	
Grade Max	*n*	36	32	X^2^: 0.1819
	1	1 (2.8%)	1 (3.1%)	
	2	6 (16.7%)	14 (43.8%)	
	3	23 (63.9%)	13 (40.6%)	
	4	4 (11.1%)	3 (9.4%)	
	5	2 (5.6%)	1 (3.1%)	
**C**				
		**Bras A: Aflibercept + sLV5FU2 ^b^**	**Bras B: sLV5FU2 ^b^**	***p*-value**
		(*n* = 21)	(*n* = 23)	
Grade Max	*n*	21	23	X^2^: 0.1353
	1	1 (4.8%)	1 (4.3%)	
	2	2 (9.5%)	7 (30.4%)	
	3	16 (76.2%)	13 (56.5%)	
	4	0 (0.0)	2 (8.7%)	
	5	2 (9.5%)	0 (0.0)	

^a^ sLV5FU2: simplified LV5FU2 regimen. ^b^ sLV5FU2: simplified LV5FU2.

**Table 5:** Progression Free Survival and Overall Survival in fluropyrimidine +/− antiangiogenic trials.

	PFS			OS		
	With Anti-Angiogenic	Without Anti-Angiogenic	*p*	With Anti-Angiogenic	Without Anti-Angiogenic	*p*
FOLFA Aflibercept +/− LV5FU2	7.4	7.3	ND	21.8	25.1	ND
MAX [8] Bevacizumab +/− capecitabine	8.5	5.7	*p* < 0.001	18.9	18.9	ND
AVEX [6] Bevacizumab +/− capecitabine	9.1	5.1	*p* < 0.0001	20.7	16.8	*p* = 0.18
XELAVIRI [17] Bevacizumab + mixed	8	ND	-	22	ND	-
FFCD 2001-02 [27] LV5FU2	ND	5.2	-	ND	14.2	-
FOCUS [28] LV5FU2	ND	6.3	-	ND	13.9	-

ND: not done. -: impossible comparison.

## Data Availability

The data that support the findings of this study are available from the FFCD upon reasonable request. Contact Cecile Girault, Administrative Manager, Fédération Francophone de Cancérologie Digestive (FFCD), Faculté de Médecine, 7, Boulevard Jeanne d’Arc, BP 87900, 21079 Dijon Cedex, France. E-mail: cecile.girault@u-bourgogne.fr.

## Supplementary Materials

The following supporting information can be downloaded at: https://www.mdpi.com/article/10.3390/cancers16081515/s1. Table S1. Inclusion and exclusion criteria.
