# Supplementary material for: First-Line LV5FU2 with or without Aflibercept in Patients with Non-Resectable Metastatic Colorectal Cancer: A Randomized Phase II Trial (PRODIGE 25-FFCD-FOLFA)
Source: Cancers (Basel). 2024 Apr 16;16(8):1515. doi: 10.3390/cancers16081515 (PMC11049283; doi:10.3390/cancers16081515)
Supplement: Supplementary file 1 [file cancers-16-01515-s001.zip › cancers-2959715-supplementary.pdf]

Table S1. Inclusion and exclusion criteria.

**Inclusion criteria**	- Age ≥ 65<br>- General condition WHO ≤ 2<br>- Metastatic rectal or colon adenocarcinoma, histologically-proven on the primary tumour or a metastasis<br>- Metastases non-resectable and/or patient inoperable<br>- Metastases not or little symptomatic<br>- At least one measurable target according to RECIST v1.1 criteria, not previously irradiated<br>- No previous treatment of the metastatic disease. Previous chemotherapy in an adjuvant situation completed 6 months or more before diagnosis of the metastasis is authorized<br>- Adequate biological examination: Hb ≥ 9 g/dl, polynuclear neutrophils ≥ 1,500/mm^3, creatinine clearance > 50 mL/mn (Cockroft and Gault formula), platelets ≥ 100,000/mm^3, total bilirubin ≤ 1.5 x UNL, creatininemia < 1.5 x UNL, ALP < 5 x UNL, AST and ALT ≤ 5 x UNL, GGT < 5 x UNL<br>- Proteinuria (strip) < 2+; if ≥ 2, test proteinuria over 24 hours which must be ≤ 1 g.<br>- Patients treated with anticoagulants (coumadin, warfarin) can be included if the INR can be closely monitored. A change in anticoagulant treatment for low molecular weight heparin is preferable in order to respect indications.<br>- Central genotyping of thymidylate synthase (TS) in blood DNA<br>- Informed consent signed
**Exclusion criteria**	- Patients with in situ primary tumour, and presenting clinical symptoms (occlusion, haemorrhage)<br>- Macronodular peritoneal carcinomatosis (risk of perforation)<br>- Cerebral metastases<br>- Uncontrolled hypercalcemia<br>- Uncontrolled hypertension (SBP > 150 mmHg and DBP > 100 mmHg) or history of hypertensive attack or hypertensive encephalopathy<br>- Any uncontrolled progressive disease over the past 6 months: hepatic insufficiency, renal insufficiency, respiratory insufficiency<br>- Subsequent complications in the 6 months prior to inclusion: myocardial infarction unstable/severe angina, coronary artery bypass, congestive cardiac insufficiency NYHA III or IV, stoke or transient ischemic attack<br>- The following conditions in the 3 months prior to inclusion: Grade 3 or 4 gastrointestinal, treatment-resistant peptic ulcer, ulcerative esophagitis or gastritis, infectious or inflammatory bowel disease, diverticulitis, pulmonary embolism or other uncontrolled thromboembolic event, unconsolidated bone fractures<br>- Major surgery during the 28 days preceding the start of treatment<br>- Known acquired immune deficiency syndrome (AIDS related illnesses) or known HIV infection requiring antiretroviral therapy<br>- Anti-cancer treatments other than the trial treatments (chemotherapy, targeted therapy, immunotherapy)<br>- History of haematological malignancies or cancer except those treated for more than 5 years and considered cured, in situ carcinomas of the cervix and skin cancers treated (melanoma excluded)<br>- Any contraindication to the treatments used in the trial<br>- Deficiency of DPD<br>- Patient treated with new oral anticoagulants (such as rivaroxaban XARELTO®, apixaban ELIQUIS®, dabigatran PRADAXA®) except if relayed by K antivitamin<br>- Pregnant or breast-feeding woman, no effective contraception in patients of child-bearing age<br>- Impossibility of undergoing medical monitoring during the trial for geographic, social or psychological reasons
